# Round the Hospitals

**Published:** 1919-08-30

**Authors:** 


### ROUND THE HOSPITALS.

The visit of the Prince of Wales to the Convent of the Ursulines at Quebec is one of those historic events which are very simple and pleasant in the doing. The great door of this convent is opened only for members of the Royal Family and their representatives the Governor-General and the Lieutenant-Governor. The nuns, nearly 100 in number, are strictly secluded, and their welcome of the Prince was one of the rare events which stir their lives to a perception of the world outside their convent walls. A photograph was taken of the Prince in uniform surrounded by the nuns. Here the wounded soldiers of Wolfe were tended, and the skull of the celebrated Montcalm, who was buried in the chapel in a hole made by a cannon-ball in the wall, is a treasured possession. No doubt the Prince will have an opportunity also of seeing in the course of his tour some of the admirable hospitals and schools for nurses for which Canada is noted.

The *Times* published on August 21 a further instalment of Sir Douglas Haig's despatch of March 16, 1919, submitting names worthy of special mention in the Nursing Services. The names

**ROUND THE HOSPITALS**—(*continued*).

comprise matrons and sisters in Q.A.I.M.N.S. and Reserve, the T.F.N.S., and the Civil Hospital Reserve. Many members of the Voluntary Aid Detachments and several among the F.A.N.Y. and Almeric Paget Military Massage Corps received mention. We note, too, the names of various sisters and nurses attached to the British Red Cross Society and of civilians who rendered special aid. Among the latter many will be glad to see the name of Miss F. Stuart-Innes, attached to the Convalescent Home for Nursing Sisters at Cannes.

It is much to be regretted that no permanent record in a wearable form has been devised for the individuals thus singled out for distinction by the Field-Marshal. It seems to lie within the province of the various bodies of nurses thus honoured to award a special badge to the "mentioned," with the date on which the despatch recording their names was issued. For members of the Civil Hospital Reserve it would be a graceful act on the part of the governing body of hospitals with whose interests they had identified themselves to bestow such badges. Cursorily to glance down the long list of names selected for special commendation gives no idea of the services which have earned the coveted distinction of Sir Douglas Haig's mention. It means the selection from some thousands of others, equally well qualified, of women conspicuously able and devoted in the cause of the wounded. The "well-done" of the Field-Marshal echoes very sweetly in the ears of those he has brought to notice.

A German version of Miss Edith Cavell's trial has been put forth, in which she is stated to have assisted spies to escape out of Belgium in association with Prince Reginald de Croij. The Prince has given a categorical denial to the assertion that Miss Cavell was his zealous assistant. He states that he came into association with her in trying to trace an officer who was stranded in Belgium and liable to be shot at sight, and that the Germans in those days shot anybody for anything. "At her trial she made no effort to hide what she had done to help her own countrymen. Not only was she the emblem of truthfulness, but as a woman she was quite distinct from any other woman I ever knew, in doing what she thought was best in the true interests of her country. . . . She gloried in her work, but there was no question of espionage. She helped defenceless men being hunted for their lives." The Prince's own sister, Princess Marie de Croij was captured and sentenced to ten years' imprisonment; she had served three years of this term before being liberated on the signing of the armistice. The trial of George Quien, accused of intelligence with the enemy, and among other crimes of having denounced Edith Cavell to the Germans, began on August 25, before the sixth court-martial in Paris. It is stated that he insinuated himself into Miss Cavell's confidence and was for a time an inmate of her hospital with the object of obtaining evidence against her.

An interesting illustrated account of Miss Cavell, "As a Pupil Knew Her," is given in the August number of "The Trained Nurse." The writer, Miss Vander Hock, refutes, like the Prince de Croij, with much spirit the accusation made against Miss Cavell of being a spy. As one of the probationers in the school she assisted in tending the refugees, some of whom were half crazed, many faint for food and drink. She describes Edith Cavell as she appeared to her probationers in striking words. "That there was much of the supernatural in her, everyone who came in contact with her felt. While she was with us we hardly recognised her spiritual strength, we were conscious that she was far above the things of the earth; the every-day annoyances that wear the average person, never stirred her beautiful serenity; we ourselves were not high enough to appreciate her soul."

An industry very suitable for nurses disabled from following their profession is that of fruit canning. Efforts are being made by the Board of Agriculture to extend it in districts where fruit ripening in great quantities is habitually wasted, and instruction is given in short courses on nominal terms. Very little capital is required, and two or three friends joining together in a small way could probably soon create a prosperous business. Full particulars of courses of training can be obtained from the Board of Agriculture, 72 Victoria Street, S.W. 1. Institutions with more fruit in the grounds than can be readily used would do wisely to get one of the staff trained in this profitable art and enlist volunteers for the light labours involved. There is an unfailing market for canned fruit, the outlay involved is small, and only unskilled labour is required, except for direction.

There are few villages in which some addition is not being planned to the cottages, and village folk are already putting their names down for the new dwellings, secure in the hope of better conveniences than they enjoy in their present homes. It is not too soon for the committees of nursing societies to be on the alert that they may induce the promoters of these building schemes to erect one for the district nurse. Bad housing accommodation is one of the main causes why district nursing is unpopular. The nurse needs a detached airy cottage, with bathroom and easy facilities for getting hot water, a room fitted with cupboards where she can keep her nursing appliances, a good bicycle-house, and kitchen premises planned with a view to labour saving. Old-fashioned cottages, with low ceilings and party walls through which every sound in neighbours' houses can be heard render the nurse's life more exhausting than need be.

The American Red Cross nursing sisters are rapidly being demobilised. There were nearly 1,800 under the Army Nurse Corps at the signing of the armistice, and of these more than half have been already released. American Red Cross nurses

**ROUND THE HOSPITALS**—*(continued)*.

are still serving in many countries, including France, Serbia, Montenegro, Roumania, Greece, Italy, Palestine, Russia, Germany, and Siberia, while some are still over here. The Chief Nurse of the Red Cross Commission to Siberia is Miss Anna L. Tittman, and the unit, which contains 140 nurses, is now complete and on its way to the seat of action. The Red Cross Bureau of Information is doing work in New York similar to that performed by the Nurses' Demobilisation Bureau in Curzon Street, in finding "jobs for nurses and nurses for jobs."

---

ONE of our contemporaries on the other side of the Atlantic quotes with approval some remarks made in these columns some week ago on the "Process of Wearing Out Matrons Before Their Time," and we gather that this deplorable and time-honoured custom is in full swing both in the United States and in Canada. We hear of a goodly number of capable women superintendents overseas broken down years before their time in hospitals which did not appreciate them enough to give additional assistance or more frequent opportunities of getting away. "When they can no longer endure the fierce strain, they are presented perhaps with a wrist-watch, a travelling-bag, or a clock, and a set of resolutions to express appreciation." Much is being done in every country to ease the strain on sisters and probationer nurses, but no one has so far proposed an eight-hour or even a twelve-hour limit for matrons. The onus of seeing that they have adequate assistance and relief must rest on the nursing committee of management.

---

CANADIAN nursing sisters are not yet prepared to accept the ruling of an eight-hour day. One of the Labour members moved an amendment to the Minimum Wage Act with the aim of including pupil nurses in training-schools in the restrictions contemplated in working hours. The Manitoba Association of Graduate Nurses, together with other graduate associations in the Province, protested against the proposed legislation, with the result that the amendment, so far as regards student nurses, was withdrawn. Again at the Convention of the Canadian National Association of Trained Nurses the matter of the eight-hour day for graduate nurses on special duty was discussed, and the Association put on record its disapproval of the system. They went on to declare that no nurse should be allowed on duty *more than twelve hours*, a decision which as manifestly condoning so great a breach of humanity and common-sense seems to us deplorable. What safety or comfort can patients derive from the ministrations of women worn down in body and mind by such excessive toil?

---

THE referendum was used with useful results lately in South Australia when the question of increased fees for private nurses came before the annual meeting of that branch of the Australasian Trained Nurses Association. The questions submitted were: (1) That nursing fees remain as they are. (2) That the nurse's fees be increased to £3 3s. a week (the only extra being steamer, coach, or train fare). The votes in favour of the increase totalled 122; those for the fees to remain as before, 41. A majority of 81 being in favour of the increase the higher rate was adopted. We hope to see the referendum largely used as time goes on for settling debatable matters. It tends as nothing else can do to make individual members of nursing societies realise their responsibilities to other members as well as to the profession. It quickens interest in the corporate decisions, and aids materially in solving knotty problems without friction.

---

AMONG the war memorials proposed in South Africa is a university on the Witwatersrand, with Chairs for Hebrew, Music, Medicine, and Domestic Science. It has been urged that if such a memorial is to be truly representative it ought to include facilities for giving higher education to nurses. To institute a Chair for Nursing and Social Science would be a worthy memorial to the devoted South African nursing sisters. If this be carried through it will not be the first time that the Overseas Dominions have gone ahead of the Old Country and achieved reforms while over here we were content to talk about them. Miss Mary Barker, who writes in the *South African Nursing Record* to urge the importance of giving nurses a place in the "great hall of learning," has, we trust, a large following.

---

OF the many odds and ends which collectively go to make up the fighting man's equipment, the least of the unconsidered trifles would seem to be a cretonne dorothy bag. Yet this modest but useful trifle became a very important item in the soldier's kit. It was issued to him when he came wounded or sick into the dressing station or hospital, and in it was put all his most cherished personal possessions, his letters, photographs, passbook, and money, and through the various transportations from line to base or home, no matter what else might be lost, so long as his small bag with its treasured possessions was safe, he was more or less content. Now the activity of the hospital bag is to cease, for its work is done and mission accomplished. Only those behind the scenes can fully realise the extent of the work so ably carried out by Lady Smith-Dorrien and her many helpers. The number of these bags distributed to our men in every theatre of war was 1,705,360, and to the wounded of our Allies 125,934, and it will be seen how great was the quantity of material, the cost and the time required for the production of such a number. The British Red Cross, whose needs were supplied by Lady Smith-Dorrien, contributed generous sums of money toward the cost of material, and one benefactor made a free gift of 1,055,000 yards of tape. To the Hospital Bag Fund there now stands a cash credit of £3,584 16s. 11d., and

**ROUND THE HOSPITALS**—*(continued)*.

it has been decided to hand this sum to the British Red Cross Society, so that they may decide upon some useful purpose on which it may be spent.

NOTHING does better service in stimulating public opinion than the exhibitions and conferences held every summer in London and devoted to nursing and midwifery interests. We learn, therefore, with much pleasure that a Nursing, Midwifery and Welfare Exhibition and Conference is to be held at Manchester, in the Free Trade Hall, early in September, and we trust that, like its London precursor, it will prove so successful as to become an annual fixture. All the worst conditions of overcrowding, ignorance, and neglect are found in the manufacturing districts in the North and Midlands, and public attention is best fixed on these evils by illustrating the methods through which they can be combated. The matters to be dealt with at the conferences include child welfare, motherhood, personal hygiene, etc., while the problem of extirpating venereal disease is the subject of one meeting. The exhibition will be open for a week from September 4. Nurses in the South will, no doubt, find a good deal to learn from the zealous promoters of the Manchester meetings.

EXCEPTIONAL recognition has been accorded by the Committee of the Austin Hospital for Incurables, Melbourne, of the meritorious services of Sister Ida Dawson, who is regarded as the "Star Nurse" of that Institution. She has constantly under her care 40 to 50 advanced cases of cancer, and last month the Secretary and Superintendent, Mr. W. J. G. Turner, was directed by the Committee to hand Miss Dawson a cheque for £25 "as a mark of their admiration of the extremely efficient and thorough manner" in which she carried out her duties. "The poor sufferers love you as you love them" is a phrase in the letter in which the Committee tenders to Miss Dawson an assurance of its gratitude and appreciation. It is good to know that devotion to duty in this difficult branch of nursing meets with such hearty appreciation on the other side of the world.

FOOD prospects are not particularly promising for large households this winter. Large buyers, however, suffer less from the profiteer than small ones, and institutions for the sick can usually command consideration from reputable tradesmen, both as old and profitable customers, and because of their appeal to what is best in human nature. It is one of the first duties of the caterer to cultivate the right attitude towards the charity among those who supply its needs and where this is successfully done, dealers will freely use their knowledge and opportunities for its advantage. More might be done in the Provinces to get into touch with market gardens and farms for the encouragement of a direct supply, and now is the time to hunt out producers and arrange to take over whatever they can furnish, potatoes, eggs, etc., without the intervention of the middleman. Hospital managers should not disdain in this respect to take a lesson from the hotel-keepers.

LUXURIES of every description continue to go up in price and institution fare tends to become more than ordinarily monotonous. We recommend the housekeeper to take steps beforehand for supplying those little relishes which convert tasteless dish to a delicacy and lend zest to a bare supper table. Dishes prepared with vinegar are very acceptable to most people, and although the process of making pickles which will keep many months is too tedious to be recommended, it requires but little time to pickle young onions, sour apples, and fresh cucumbers, or celery, for present use. They should be sliced in equal quantities and placed in an unglazed, earthen gallon jar, which must be filled with the pickle. A good pickling mixture is composed of a tablespoonful of salt, two teaspoonfuls of cayenne pepper, a little sherry if procurable, a small bottle of soy or other savoury sauce, and as much brown vinegar as the jar will hold. Water may be mixed with the vinegar if economy is desired. Pickled fish is a very useful dish in hot weather. The fish must be cut into neat pieces and packed in a stone jar. The pickle is made of a quart of vinegar (diluted if preferred), an ounce of salt, half a tablespoonful of horse-radish, half a dozen cloves, a little allspice, one or two bay leaves, and half a teaspoonful of celery-seed, cayenne and black pepper. Boil the pickle, pour enough over the fish to cover it, tie the jar down and cook in a very slow oven till done. It should be allowed to get cold before removing the cover.

THE Kent and Canterbury Hospital is appealing for books and papers for its military and other patients. The need is one which can only be met by organisation. If a receptacle for stray books and papers is set within easy reach of the public, many persons will get into the habit of dropping in their superfluous magazines who would not ever find time to do up a parcel and despatch it. A well-placed box outside the station or where the trams stop, with an attractive notice on it, will not only beguile travellers to bestow many gifts of this kind, but will prove a useful advertisement for the charity. Another box of the kind should always be kept at the hospital gates.

MANY are the impediments which the nurse encounters in this country in her efforts to introduce fresh air into her patients' rooms, but at least she is not hampered by their fear that diseases are borne on the night winds and are distributed by evil spirits who get access through open windows. This is the prevailing belief in Montenegro, and, as tuberculosis is rife, the inhabitants are in a parlous state. The American Red Cross has a devoted band of nursing sisters in Roumania, and much may be expected from their propaganda and personal example.

